# Acute Kaempferol Stimulation Induces AKT Phosphorylation in HepG2 Cells

**DOI:** 10.3390/life14060764

**Published:** 2024-06-15

**Authors:** Beatriz Santana-Lima, Lucas Humberto Zimmermann Belaunde, Karine Damaceno de Souza, Matheus Elias Rosa, Jose Eduardo de Carvalho, Joel Machado-Jr, Maria Isabel Cardoso Alonso-Vale, Luciano Caseli, Daniela Gonçales Galasse Rando, Luciana Chagas Caperuto

**Affiliations:** 1 Programa de Pós-Graduação em Biologia Química, Instituto de Ciências Ambientais, Químicas e Farmacêuticas—ICAQF, Universidade Federal de São Paulo, Diadema 09913-030, SP, Brazil; beatriz.santana13@unifesp.br (B.S.-L.);; 2 Programa de Pós-Graduação em Química—Ciência e Tecnologia da Sustentabilidade, Instituto de Ciências Ambientais, Químicas e Farmacêuticas—ICAQF, Universidade Federal de São Paulo, Diadema 09913-030, SP, Brazil; 3 Departamento de Biologia e Ecologia Evolutiva, Instituto de Ciências Ambientais, Químicas e Farmacêuticas—ICAQF, Universidade Federal de São Paulo, Diadema 09913-030, SP, Brazil; 4 Departamento de Ciências Biológicas, Instituto de Ciências Ambientais, Químicas e Farmacêuticas—ICAQF, Universidade Federal de São Paulo, Diadema 09913-030, SP, Brazil; 5 Departamento de Química, Instituto de Ciências Ambientais, Químicas e Farmacêuticas—ICAQF, Universidade Federal de São Paulo, Diadema 09913-030, SP, Brazil; 6 Departamento de Ciências Farmacêuticas, Instituto de Ciências Ambientais, Químicas e Farmacêuticas—ICAQF, Universidade Federal de São Paulo, Diadema 09913-030, SP, Brazil

**Keywords:** type 2 diabetes mellitus, flavonoids, insulin signaling, liver cells

## Abstract

Type 2 diabetes mellitus (T2DM) stands as a prevalent global public health issue caused by deficiencies in the action of insulin and/or insulin production. In the liver, insulin plays an important role by inhibiting hepatic glucose production and stimulating glycogen storage, thereby contributing to blood glucose regulation. Kaempferitrin (KP) and kaempferol (KM), flavonoids found in *Bauhinia forficata*, exhibit insulin-mimetic properties, showing promise in managing T2DM. In this study, we aimed to assess the potential of these compounds in modulating the insulin signaling pathway and/or glucose metabolism. Cell viability assays confirmed the non-cytotoxic nature of both compounds toward HepG2 cells at the concentrations and times evaluated. Theoretical molecular docking studies revealed that KM had the best docking pose with the IR β subunit when compared to the KP. Moreover, Langmuir monolayer evaluation indicated molecular incorporation for both KM and KP. Specifically, KM exhibited the capability to increase AKT phosphorylation, a key kinase in insulin signaling, regardless of insulin receptor (IR) activation. Notably, KM showed an additional synergistic effect with insulin in activating AKT. In conclusion, our findings suggest the potential of KM as a promising compound for stimulating AKT activation, thereby influencing energy metabolism in T2DM.

## 1. Introduction

Diabetes stands as a chronic non-communicable disease characterized by inadequate insulin production or utilization, often leading to hyperglycemia [1] Globally, diabetes poses a significant public health challenge. In 2021, among 5.1 billion adults aged 20 to 70, there were 536.6 million cases of diabetes, representing a prevalence of 10.5% [1].

**Figure 1:**
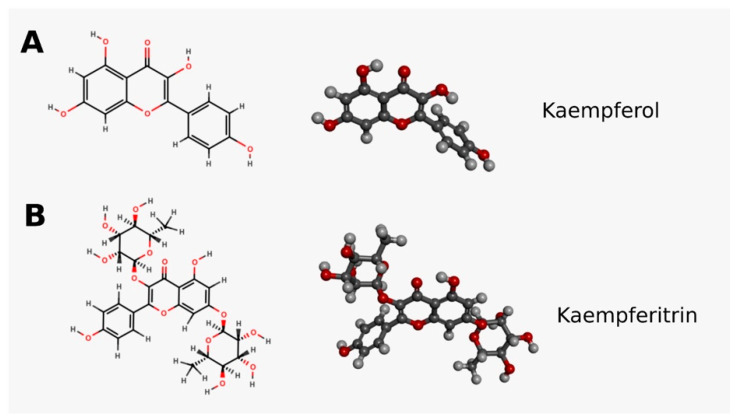
Chemical structure of kaempferol (KM) (**A**) and kaempferitrin (KP) (**B**). MarvinSketch20.11 and Biovia Discovery Studio Visualizer v.21.1.0.20298.

Of many strategies for treating Type 2 diabetes mellitus (T2DM) [2], phytotherapy has emerged as a prominent avenue, employing diverse medicinal plants for therapeutic intervention. Among these herbal medicines, *Bauhinia forficata Link* (Class: Magnoliopsida (Dicotiledonae); Order: Fabales; Family: Caesalpiniaceae (Leguminosae Caesalpinioideae)) stands out for its impact on glucose and lipid metabolism due to its hypoglycemic effect and its antioxidant properties [2]. Kaempferol (KM) and kaempferitrin (KP) are compounds found in *B. forficata* and are known for their antidiabetic and hypoglycemic effects. Both compounds have different chemical structures. KM is a tetrahydroxyflavone with four hydroxyl groups located in positions 3, 5, 7, and 4’. KP (kaempferol 3,7-di-O-alpha-L-rhamnoside) is a glycosyloxyflavone, which is basically the molecule of KM linked to alpha-L-rhamnopyranosyl residues in positions 3 and 7 through glycosidic bonds. KM is a tetrahydroxyflavone with four hydroxyl groups located in positions 3, 5, 7, and 4’ [3] (Figure 1A,B).

Oral administration of KM in diabetic Wistar rats significantly improved plasma glucose, insulin, glycated hemoglobin (HbA1c), and enzymes related to carbohydrate metabolism, including glucokinase [4]. The improvement in peripheral insulin sensitivity resulting from KM administration may be due to the fact that this compound reduces the hepatic inflammation observed in animals subjected to STZ diabetes [5]. In in vitro experiments, incubation of soleus muscle with a glycosylated derivative of KM, kaempferol-3-neohesperidoside, stimulates glucose uptake, probably due to its action on signaling pathways involving phosphatidylinositol 3-kinase (PI3K) and protein kinase C (PKC) proteins, since, when these pathways were inhibited, the effect was completely inhibited [6]. Regarding KP, there are few studies that evaluate the effects of this compound on the insulin signaling pathway. A single oral dose of KP in diabetic rats significantly decreased glycemia and increased the muscle glucose uptake in vitro [7]. Cazarolli et al., (2013), suggested the involvement of mediators of insulin signaling, such as the insulin receptor (IR), PI3K, PKC, mitogen-activated protein kinases (MAPK), and GLUT4 translocation in KP’s effect on glycemic homeostasis, particularly emphasizing its potential action in skeletal muscle [8]. However, further understanding of the mechanisms underlying the efficacy of medicinal plants such as KP and KM is essential to mitigate the metabolic and glycemic complications associated with T2DM [9]. To our knowledge, there are no studies in the literature comparing the two compounds directly.

In this study, we investigated the impact of KP and KM on modulating insulin signaling in liver cells (HepG2), focusing on their interaction with the IR and specifically targeting the ATP recognition site. Additionally, we explored the potential interaction of these compounds with cell membrane models using Langmuir monolayers, a well-established method for assessing interactions between bioactive molecules [10,11,12], such as KP and KM, and lipidic interfaces. Understanding the initial interaction of these compounds with the plasma membrane’s external leaflet is critical in determining their subsequent interactions within the cytoplasm.

## 2. Material and Methods

### 2.1. Culture of HepG2 Cells

Human hepatocarcinoma (HepG2) cells, purchased from Cell Bank (Rio de Janeiro, Brazil), were cultivated with DMEM culture medium (Dulbecco’s modified eagle medium; Gibco, Thermo Fisher Scientific, Waltham, Massachusetts, USA) supplemented with 5.5 mM glucose, 10% fetal bovine serum, 100 U/mL of penicillin, and 0.1 mg/mL of streptomycin, in an atmosphere of 5% CO_2_, at 37 °C. HepG2 cells are a suitable model to evaluate the insulin signaling pathway and gluconeogenesis [13,14]. Kaempferitrin (KP; SML1853-1MG ≥ 97% (NMR); Merck KGaA, Darmstadt, Germany) was used at concentrations ranging from 0.1 µM to 50 µM [15,16], while kaempferol (KM; 60010-25MG ≥ 97.0% (HPLC); Merck KGaA, Darmstadt, Germany) was used from 0.1 µM to 100 µM [17]. Both compounds were diluted from a 10 mM stock solution in ethanol (Merk KGaA, Darmstadt, Germany) with DMEM medium. This study was approved by the Committee for Ethics in Research (# 2352150820).

### 2.2. Cell Viability Assay

The cells were incubated in 96-well plates at a density of 1 × 10^5^ cells/well for 48 h, followed by fetal bovine serum deprivation for 18 h, and subsequently treated with KP or KM, at concentrations 0.1, 1, 10, 20, and 50 μM and 0.1, 1, 10, 20, 50, 80, and 100 μM, respectively, for 24 or 72 h. After this period, the cells were incubated in DMEM 5.5 mM glucose medium supplemented with 100 U/mL of penicillin and 0.1 mg/mL of streptomycin, and with 3-methyl-[4-5-dimethylthiazol-2-yl bromide]-2.5 diphenyltetrazolium (MTT; Molecular Probes by Life Technologies, Merk KGaA, Darmstadt, Germany—50 µg/well), for 4 h at 37 °C in an atmosphere with 95% O_2_ and 5% CO_2_. Formazan crystals were dissolved using a solubilization solution (DMSO and 50%/50% isopropyl alcohol). The absorbance was measured using a spectrophotometer (Epoch TM, Bio-Tek, Winooski, VT, USA) at a wavelength of 570 nm. A positive control with dimethyl sulfoxide 30% (DMSO; Synth, Brazil) and a vehicle control (1%) were also included.

### 2.3. Protein Extraction and Western Blotting Analysis

The cells were incubated in 6-well plates with a density of 6 × 10^5^ cells/well for 48 h. Then, fetal bovine serum deprivation was performed for 24 h before the initiation of treatments. Human insulin (Novolin R; Novo Nordisk; São Paulo, Brazil) was used at a concentration of 100 nM [18] for 5, 10, 15, and 20 min to determine the optimal time for assessing AKT phosphorylation. To analyze protein expression and phosphorylation levels in the insulin signaling pathway, KP 10 μM and KM 10 μM [19,20,21] were employed. The cells were lysed using RIPA lysis buffer (20 mM Tris-HCl pH 7.4, 1 mM EDTA pH 8, 10% glycerol, 20 mM sodium fluoride, 30 mM sodium pyrophosphate, 0.2% SDS, 1 mM sodium orthovanadate, and 5 μM aprotinin) and centrifuged at 15,000× *g* at 4 °C for 20 min to collect the supernatant. The protein content was determined by spectrophotometry with Bradford reagent (Bio-Rad protein assay; Bio-Rad, Hercules, CA, USA). Protein samples were separated on a gel and transferred to a nitrocellulose membrane using a wet transfer system (Bio-Rad, CA, USA). Following blocking (5% BSA), membranes were washed with TBST, incubated with the primary antibodies, and kept at 4 °C overnight. Then, they were washed, incubated with the specific secondary antibody, and processed for image acquisition using the “UVI Band” program in the Uvitec Cambridge equipment (Warwickshire, UK). Band intensities were quantified using Image J (NIH). The primary antibodies used were all from Cell Signaling Technology (Danvers, MA, USA): IRβ (code: #23413, 1:1000), pTYR (code: #9416,1:2000), AKT (code: #9272, 1:1000), pAKT1/2/3 (code: #9271, 1:1000), ERK1/2 (code: #9102, 1:1000), pERK1/2 (code: #9101, 1:1000). *β*-actin primary antibody for constitutive analysis was from Sigma-Aldrich (Saint Louis, USA; code: #A5316, 1:10,000). The secondaries used were anti-rabbit (code: #7074, 1:60,000) or anti-mouse (code: #7076, 1:30,000), both from Cell Signaling Technology (Danvers, MA, USA).

After revealing the phosphorylated proteins, stripping was carried out (200 mM glycine, 3.4 mM SDS, 1% Tween 20, and pH 2.2) and revealed using an antibody to evaluate the total protein. For some proteins (AKT and ERK), a new stripping was performed to reveal the constitutive protein (*β*-actin). In Figure 7, the representative images of total ERK (Figure 7D–F) are the same as those used in Figure 8 (Figure 8D–F). In Figure 8, the representative images of *β*-actin (Figure 8C,F) are the same.

### 2.4. Molecular Docking Studies

The *Homo sapiens* insulin receptor tyrosine kinase region crystal structure (PDB ID 3EKK, 2.10 Å) was downloaded from the RCSB Protein Data Bank, and the 3D structures of KM (PubChem CID #5280863) and KP (PubChem CID #5486199) were downloaded from the NCBI PubChem. Both compounds were used to perform the semi-rigid docking analyses employing a setup protocol obtained from redocking studies considering the original co-crystallized inhibitor as the “test” ligand. The three-dimensional and energetic optimization of the small molecules was performed employing the Gaussian 09W software up to 0.01 kcal/mol and the proteins were preprocessed by hydrogen addition to the pdb file. All original water molecules were kept.

Gold 2020.3.0 Software^®^ (GOLD Suite Intuitive Protein–Ligand Docking Package, Cambridge, UK) was selected, then adjusted to run sets of 10 answers each run considering a centroid (X, Y, Z = −3.6865, 18.8945, −14.1345) and a cavity of 10 Å around this point as the reference for the docking of the KP and KM. Ten runs were performed to complete a set of 100 resulting poses which were ordered following the Goldscore scoring function.

A consensus analysis among the obtained poses was used to choose the most prevalent ones, considering a frequency greater than or equal to 70% as ideal for interactions and visual analysis [22,23,24,25].

### 2.5. Langmuir Monolayers

KP and KM were dissolved in ethanol (Merk KGaA, Darmstadt, Germany), resulting in 0.5 mg/mL concentrations. 1,2-dipalmitoyl-sn-glycero-3-phosphocholine (DPPC), a lipid frequently found in erythrocyte membranes [26] and popularly employed as cellular membranes as the first model in Langmuir monolayers [27,28,29], was purchased from Sigma-Aldrich and dissolved in CHCl_3_ (Labynth) to yield 0.5 mg/mL solutions. A Langmuir trough (model mini; KSV Instruments, Helsinki, Finland) was previously cleaned with handkerchiefs containing ethanol and CHCl_3_ and filled with purified water (resistivity 18.2 MΩ cm, surface tension 72.0, and pH 6.2 at 25 °C). The trough and other equipment pieces coupled with it were kept in a clean room at a constant temperature (25 ± 1 °C). KP and KM at selected molar proportions were cospread with DPPC on the air–water. At least 10 min passed for solvent evaporation, and then monolayers were compressed using symmetric barriers at a rate of 2 Å^2^ molecule^−1^ min^−1^.

Surface pressure (π)–area (A) isotherms were obtained by measuring the water surface tension decrease with a Wilhelmy filter paper intercepting the interface at the center of the trough. Surface compression modulus (K) values were obtained through the π-A isotherms by applying the equation K = −(∂π/∂A)_T_, as suggested by Davies and Rideal [30]. Stability curves were obtained by compressing the monolayers to the desired surface pressure (30 mN/m) and following the π variation with time with a constant film area. Rheological parameters were obtained with the oscillating barrier method [31]: the monolayer was compressed to 30 mN/m and left to keep the surface pressure constant, with the barriers going back and forth for 10 min to stabilize the monolayer. Then, the monolayer was subjected to 10 short cycles of compression–expansion (2% of area decrease–increase at a 2 mHz frequency), and the viscoelastic parameters were calculated based on the average surface pressure variation to the area oscillation and the phase angle between the area and surface pressure sinusoidal curves. Brewster Angle Microscopy (BAM) was employed to characterize the morphology of the monolayers with an apparatus from KSV-Instruments (Helsinki, Finland) positioned in the center of the trough. The monolayer was compressed up to 30 mN/m and permitted to relax for at least 60 min to follow changes in the morphology. For each image presented here, the most representative images are shown, which means that if some isolated agglomerates were found, probably related to impurities, such images were discarded. Each experiment was repeated at least three times to ensure the reproducibility of the results. The most representative curves or images are shown.

### 2.6. Statistical Analysis

The experiments were carried out with 4 to 10 different passages. Data are expressed as the mean ± standard error of the mean (SEM). Homogeneity analysis was performed by Grubbs (5%) to exclude outliers. Normality analysis was performed using the Shapiro–Wilk test. Therefore, an ANOVA was used for all variables using treatment as a fixed factor, followed by Tukey’s post-test. Analyses were performed using the GraphPad Prism 9.0 program, considering a significance level of 5% (*p* < 0.05) and a trend of 10% (*p* < 0.1).

## 3. Results

### 3.1. KP and KM Do Not Interfere with Cell Viability

**Figure 2:**
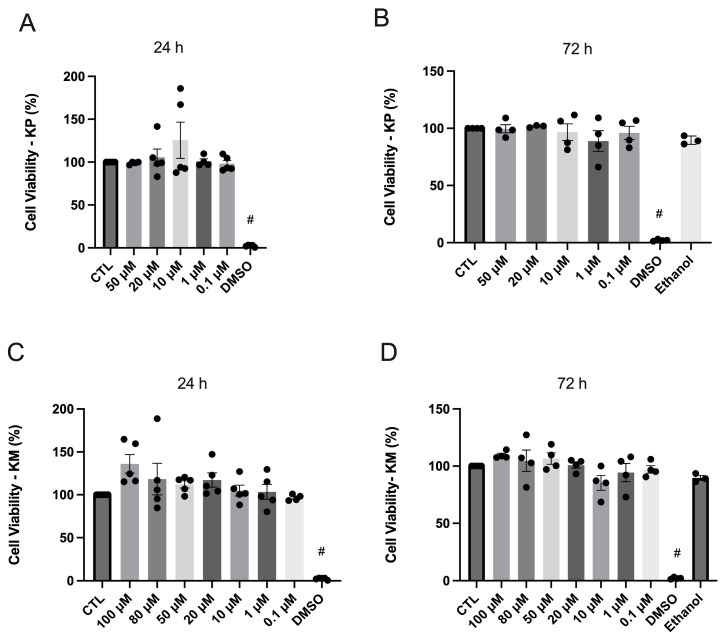
MTT assay. (**A**,**B**) Groups: control (CTL), administration of KP at concentrations 50, 20, 10, 1, and 0.1 μM, and 30% DMSO after incubation for 24 h and 72 h in HepG2 cells. *n* = 3–5. (**C**,**D**) Groups: control (CTL), administration of KM at concentrations 100, 80, 50, 20, 10, 1, and 0.1 μM, and 30% DMSO after incubation for 24 h and 72 h in HepG2 cells. *n* = 4–5. # DMSO 30% vs. all other groups; *p* < 0.0001. The dots are the representations of each *n* in the experiment.

The 24 h and 72 h treatments using kaempferitrin (KP) within the concentration range of 0.1 μM to 50 μM (Figure 2A,B, respectively), as well as kaempferol (KM), within the concentration range of 0.1 μM to 100 μM (Figure 2C,D, respectively), exhibited no cytotoxic effect on HepG2 cells. Additionally, in the 72 h treatment, the inclusion of the ethanol administration group revealed no discernible variance compared to other groups. Notably, exposure to 30% DMSO, utilized as a positive control, resulted in a complete loss of cell viability (*p* < 0.0001).

### 3.2. KP and KM Do Not Stimulate IR Phosphorylation

**Figure 3:**
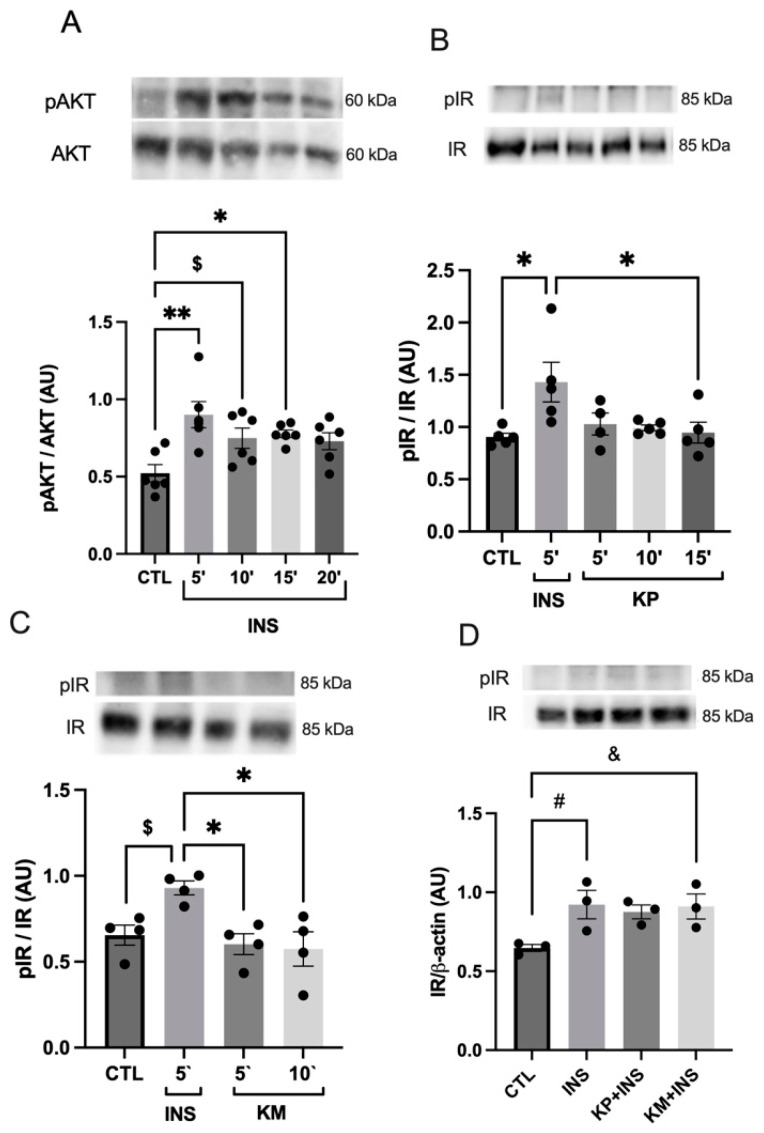
AKT and insulin receptor phosphorylation were detected by Western blotting in the HepG2 cell line. (**A**) AKT phosphorylation. Groups: control (CTL), stimulation with insulin at a concentration of 100 nM for 5, 10, 15, and 20 min in HepG2 cells. *n* = 5–6 * CTL vs. 15 min; ** CTL vs. 5 min; $ CTL vs. 10 min (*p* = 0.0890). (**B**) Insulin receptor phosphorylation. Groups: control (CTL), stimulation with insulin at a concentration of 100 nM for 5 min (INS) and KP administration at a concentration of 10 μM during 5, 10, and 15 min. *n* = 4–5. * CTL vs. 5 min; * 5 vs. 15 min. (**C**) Insulin receptor phosphorylation. Groups: control (CTL), stimulation with insulin at a concentration of 100 nM for 5 min (INS) and KM administration at a concentration of 10 μM for 5 and 10 min. *n* = 4. $ CTL vs. INS (*p* = 0.0581); * INS vs. 5 min; * INS vs. 10 min. (**D**) Insulin receptor phosphorylation. Groups: control (CTL), stimulation with insulin at a concentration of 100 nM (INS); administration of KP at a concentration of 10 μM plus insulin at a concentration of 100 nM (KP + INS); administration of KM at a concentration of 10 μM plus insulin at a concentration of 100 nM (KM + INS); stimulation for 5 min. *n* = 3. # CTL vs. INS (*p* = 0.0984); and & CTL vs. KM + INS (*p* = 0.1005). The dots are the representations of each *n* in the experiment.

After assessing cell viability, the time course for insulin administration (INS) was established to pinpoint the peak of AKT phosphorylation since INS was used as a positive control. Following INS administration, a noticeable escalation in AKT phosphorylation occurs, displaying a significant difference at 5 (*p* = 0.0014; Figure 3A) and 15 min (*p* = 0.0442; Figure 3A) compared to the CTL group. When insulin was administered for 10 min, there was a trend toward an increase in the degree of AKT phosphorylation (*p* = 0.0890; Figure 3A) compared to the CTL group. However, after 20 min, no discernible differences were observed compared to all other times. Consequently, owing to the marked AKT phosphorylation observed at 5 min post-insulin administration, this time was chosen as the standard positive control for all subsequent experiments (Figure 3A).

To assess the effects of KP and KM on components of the insulin receptor signaling cascade, HepG2 cells underwent treatment with each compound, enabling the evaluation of AKT and IR phosphorylation levels. Remarkably, the results revealed an increase in IR phosphorylation at the 5 min mark following insulin exposure, showing statistically significant differences in comparison to the control (*p* = 0.0193 and *p* = 0.0581; Figure 3B,C, respectively). After KM or KP administration, the levels of IR phosphorylation showed no significant deviation across the assessed time points compared to the CTL group. Upon evaluating the potential additive effect of KM and KP, a tendency towards increased IR phosphorylation was observed with insulin (*p* = 0.0984; Figure 3D) and KM and insulin combination (*p* = 0.1005; Figure 3D). However, the combination of insulin and KP revealed no significant increase in IR phosphorylation (*p* = 0.1412).

### 3.3. Molecular Docking as a Predictor of the Potential Binding of Kaempferol and Kaempferitrin to Insulin Receptor

After evaluating the effects of KM and KP on the IR phosphorylation, the possibility of interaction between these compounds and the IR through molecular docking was evaluated. To set and validate the modeling protocol, redocking studies were previously performed to find poses similar to that found in the crystal. The conditions described in the Methods section achieved such a similar pose with a frequency of 91% when compared to the ATP analog located in the kinase region of the crystal IR model. The best scoring function for this system was the Goldscore implemented in the Gold 2020.3.0 Software ^®^

KM and KP were then docked under the same conditions adjusted by the redocking studies, and the best poses according to Goldscore function were chosen for the visual analysis. The results of anchoring with kaempferol showed that this molecule could assume a main pose with 99% frequency, and the best-rated pose achieved a Goldscore of 45.0485 (solution 2 of run 3). The overall variation in the scoring values was from 40.00 to 45.05, a narrow range that could reveal the closeness of the poses. In the case of kaempferitrin, the best pose achieved a frequency of 90% with the best-scoring result of 47.0399 (solution 2 of run 5), with a variation in Goldscore values in a much larger range, from 10.00 to 50.00, however.

**Figure 4:**
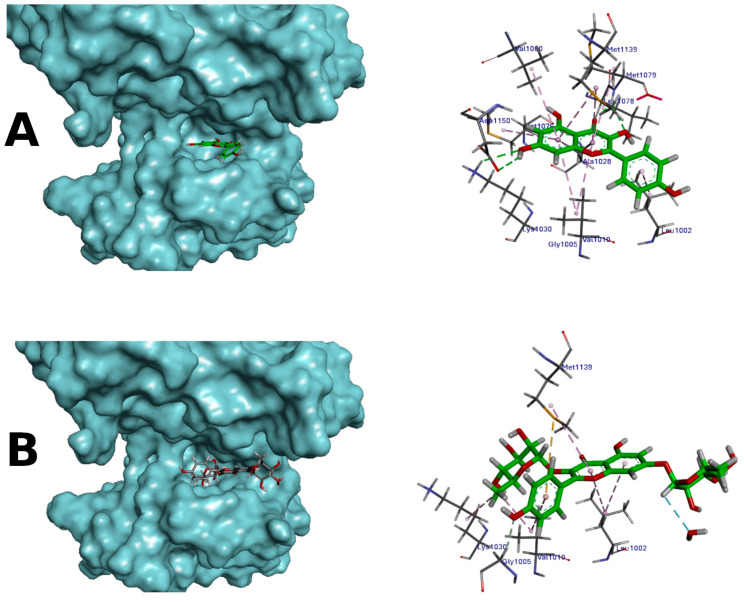
(**A**) General overview of kaempferol (KM) docked into the *β* subunit of the insulin receptor represented as a surface-covered structure and predicted binding mode of kaempferol (KM) highlighting the interaction with the cavity amino acids. Two hydrogen bonds were observed. (**B**) General overview of kaempferitrin (KP) docked into the insulin receptor kinase. Receptor represented as a surface-covered structure. Predicted binding mode of kaempferitrin (KP) highlighting the interaction with the cavity amino acids. One hydrogen bond was observed. Carbons in aquamarine, nitrogens in blue, oxygens in red, and hydrogens in white. Orange dotted lines—hydrogen bond-type interactions.

The molecular docking analysis indicated that KM has the possibility of anchoring in the binding region of the ATP site present in the *β* subunit of insulin receptor (Figure 4A,B, for KM). In the case of kaempferitrin, the high frequency of a similar pose indicates the potential for being a ligand of the IR, but the much larger range among the scoring values can also indicate that this could be a less effective ligand of the receptor (Figure 4C,D, KP). Regardless, both could displace the ATP from its recognition site in the IR, interfering with the phosphorylation processes.

### 3.4. KM and KP Display Interaction with Cell Membrane Models

**Figure 5:**
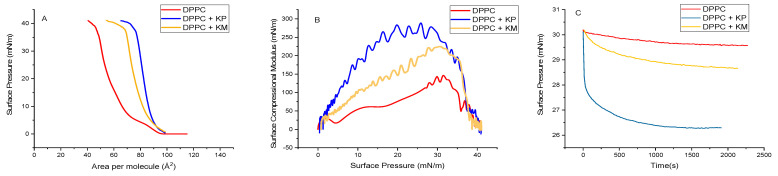
Tensiometric measurements of cell membrane models (DPPC monolayers) and their effect upon incorporating KP and KM. (**A**) surface pressure–area isotherms; (**B**) surface compressional modulus–surface pressure isotherms; and (**C**) surface pressure–time isotherms for previously compressed monolayers up to 30 mN/m, keeping the area constant.

Figure 5A illustrates the surface pressure–area isotherms in DPPC monolayers and the effect upon KP and KM incorporation. The DPPC exhibits a characteristic curve [32], showing the lift-off area at 95 Å^2^, where the liquid-expanded state was reached upon compression. A typical pseudo-plateau emerges at 5 mN/m, denoting the 2D transition between the liquid-expanded and the liquid-condensed states. Upon reaching 65 Å^2^, a sudden elevation in the curve represents the transition to the liquid-condensed phase and the collapse. It is important to note that the inclination of this state and the collapse pressure may slightly differ from other literature data due to variations in the geometry rate of compression and standardized levels of the meniscus for each experiment. With the inclusion of KP and KM, the isotherms are shifted to higher areas, indicating molecular incorporation. The shift was higher for KP due to its higher voluminous molecular structure due to the glycidic groups.

The surface compressional modulus (K), presented in Figure 5B, is directly derived from the isotherms. DPPC reached a maximum of 125 mN/m, attributed to the liquid-condensed state. Upon the introduction of KP and KM, these maxima increased, indicating a more rigid monolayer. Incorporating these molecules into the lipid structure effectively filled the geometrical imperfections in lateral packing, rendering the film less molecularly compressible. The stability of previously compressed monolayers also decreased with KP and KM. These compressed monolayers, up to 30 mN/m, were allowed to relax to reach their equilibrium pressure. While pure DPPC reached an equilibrium pressure of around 29.6 mN/m, the mixed monolayer displayed enhanced molecular rearrangements, enabling it to achieve a lower free energy value (Figure 5C). This effect is more pronounced for the more voluminous molecule (KP).

**Figure 6:**
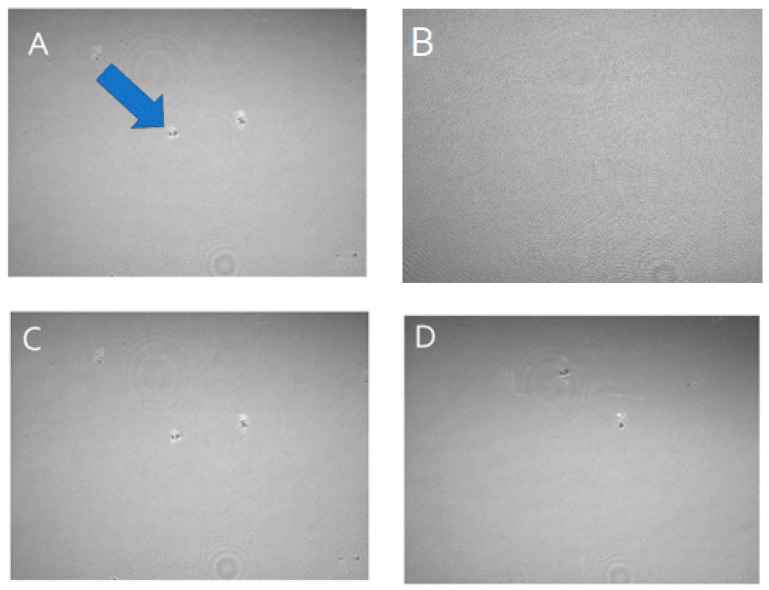
BAM images (3600 × 2400 μm) of DPPC without (**A**,**C**) and with KP (**B**) and KC (**D**) at 30 mN/m. The arrow shows an example of a common aggregate formed when the monolayer is close to the collapse point.

The relaxed monolayer, compressed at 30 mN/m and subjected to dynamic cycles of compression and expansion, revealed an unexpected effect (Table 1): the introduction of KP and KM renders the film more fluid, evident through decreased G* values. This behavior is likely attributed to molecular relaxation, allowing more possibilities for molecular rearrangements when the interface was submitted to a strain, which reduces the molecular stress. Interestingly, while the surface viscosity increases with the introduction of KP and KM, as indicated by the phase angle, these compounds increase the internal frictional force between adjacent layers, compelling movement during film deformation. Nevertheless, as there was no evidence of interfacial domain formation in BAM images (Figure 6), these alterations cannot be attributed to the potential formation of aggregates.

### 3.5. KM Has a Possible Additional Effect with Insulin in Inducing the Degree of AKT Phosphorylation

**Figure 7:**
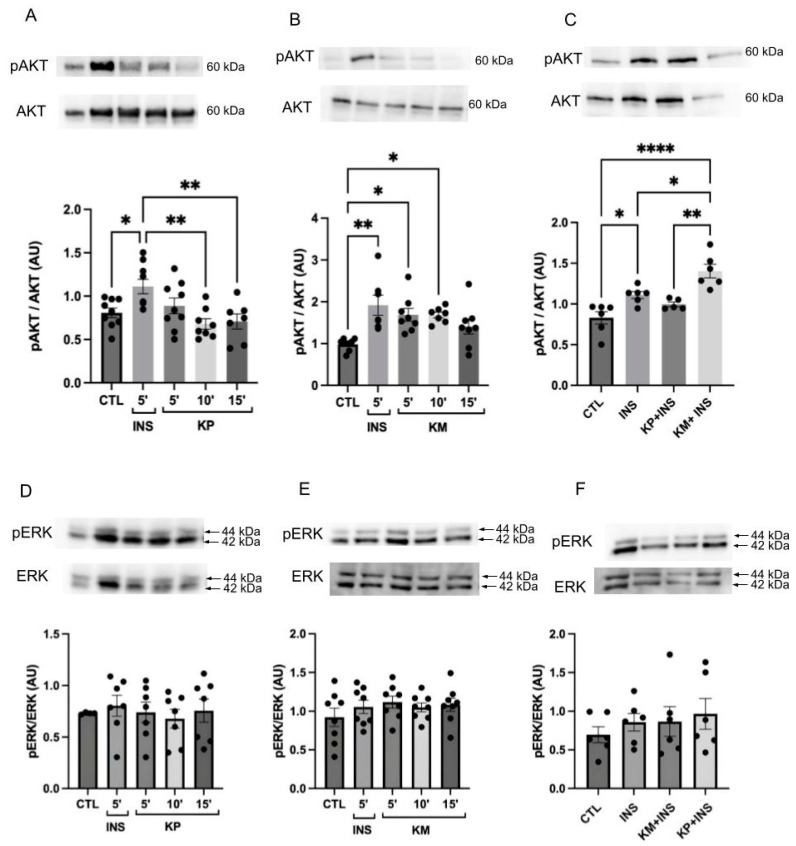
AKT and ERK phosphorylation were detected by Western blotting in the HepG2 cell line. (**A**) AKT phosphorylation. Groups: control (CTL), stimulation with insulin at a concentration of 100 nM for 5 min (INS) and KP administration at a concentration of 10 μM during 5, 10, and 15 min in HepG2 cells. *n* = 7–9 * CTL vs. INS; ** INS vs. 10 and 15 min; (**B**) AKT phosphorylation. Groups: control (CTL), stimulation with insulin at a concentration of 100 nM for 5 min (INS) and KM administration at a concentration of 10 μM during 5, 10, and 15 min in HepG2 cells. *n* = 7–8 * CTL vs. 5 min; ** CTL vs. INS and 10 min; (**C**) AKT phosphorylation. Groups: control (CTL), stimulation with insulin at a concentration of 100 nM for 5 min (INS); administration of KP at a concentration of 10 μM plus insulin at a concentration of 100 nM (KP + INS); administration of KM at a concentration of 10 μM plus insulin at a concentration of 100 nM (KM + INS). Stimulation for 5 min. *n* = 5–6 * CTL vs. INS; * INS vs. KM + INS; ** KP + INS vs. KM + INS; **** CTL vs. KM + INS (**D**) ERK phosphorylation. Groups: control (CTL), stimulation with insulin at a concentration of 100 nM for 5 min (INS) and KP administration at a concentration of 10 μM during 5, 10, and 15 min in HepG2 cells. *n* = 4–7. No statistical difference between groups. (**E**) ERK phosphorylation. Groups: control (CTL), stimulation with insulin at a concentration of 100nM for 5 min (INS) and KM administration at a concentration of 10 μM during 5, 10, and 15 min in HepG2 cells. *n* = 7. No statistical difference between groups. (**F**) ERK phosphorylation. Groups: control (CTL), stimulation with insulin at a concentration of 100 nM for 5 min (INS); administration of KP at a concentration of 10 μM plus insulin at a concentration of 100 nM (KP + INS); administration of KM at a concentration of 10 μM plus insulin at a concentration of 100 nM (KM + INS). Stimulation for 5 min. *n* = 6. No statistical difference between groups. The representative images of total AKT (**A**–**C**) are the same as those used in Figure 8 (Figure 8A–C). The representative images of total ERK (**D**–**F**) are the same as those used in Figure 8 (Figure 8D–F). The dots are the representations of each *n* in the experiment.

There was a marked increase in AKT phosphorylation following insulin administration compared to the CTL group (*p* = 0.0460, Figure 7A; *p* = 0.0013, Figure 7B; *p* = 0.0251; Figure 7C). Post-KP administration, AKT phosphorylation levels remained unaltered at all evaluated times compared to the CTL group (Figure 7A). However, treatment with 10 μM KM exhibited increased AKT phosphorylation at 5 (*p* = 0.0166; Figure 7B) and 10 min (*p* = 0.0236; Figure 7B) in the CTL group. At 15 min, phosphorylation levels remained intermediate, showing no significant difference compared to CTL and INS groups. A combined assay of KM and KP showed an additive effect on AKT phosphorylation, where the KM + INS group demonstrated elevated phosphorylation compared to the CTL group (*p* < 0.0001; Figure 7C) and the INS group (*p* = 0.0183; Figure 7C). AKT phosphorylation levels were similar between KP + INS and the CTL or INS groups. Furthermore, the KM + INS group displayed increased AKT phosphorylation compared to the KP + INS group (*p* = 0.0017; Figure 7C).

The evaluation of the MAPK pathway demonstrated no modulation in ERK1/2 phosphorylation following insulin, KP, or KM treatment across all evaluated times and conditions (Figure 7D–F).

**Figure 8:**
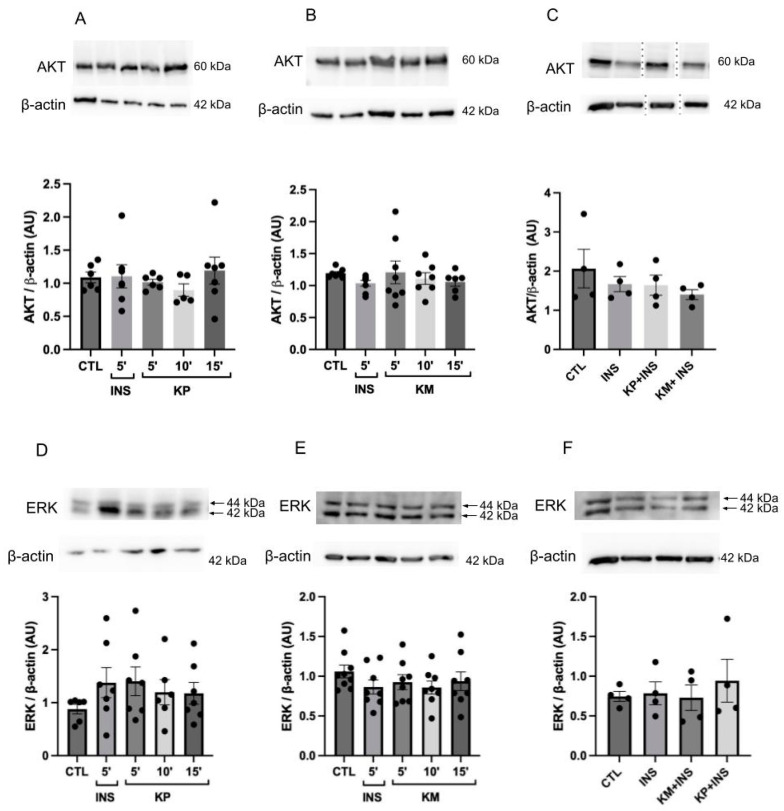
AKT and ERK expression detected by Western blotting in HepG2 cell line. (**A**,**D**) Expression of AKT and ERK, respectively. Groups: control (CTL), stimulation with insulin at a concentration of 100 nM for 5 min (INS) and KP administration at a concentration of 10 μM during 5, 10, and 15 min in HepG2 cells. *n* = 6–9. No statistical difference between groups; (**B**,**E**) Expression of AKT and ERK, respectively. Groups: control (CTL), stimulation with insulin at a concentration of 100 nM for 5 min (INS) and KM administration at a concentration of 10 μM during 5, 10, and 15 min in HepG2 cells. *n* = 6–9. No statistical difference between groups; (**C**,**F**) Expression of AKT and ERK, respectively. Groups: control (CTL), stimulation with insulin at a concentration of 100 nM for 5 min (INS); administration of KM at a concentration of 10 μM plus insulin at a concentration of 100 nM (KM + INS); administration of KP at a concentration of 10 μM plus insulin at a concentration of 100 nM (KP + INS). Stimulation for 5 min. *n* = 4. The representative images of total AKT (Figure 7A–C) are the same as those used in Figure 8 (**A**–**C**). The representative images of total ERK (Figure 7D–F) are the same as those used in Figure 8 (**D**–**F**). The representative images of *β*-actin (**A** and **D**, **B** and **E** and **C** and **F**) are the same. The dots are the representations of each *n* in the experiment.

Assessing the total protein expression for AKT and ERK showed no statistically significant differences among the treated groups (Figure 8A–F).

## 4. Discussion

Molecular docking serves as an invaluable in silico tool that significantly contributes to drug development and discovery. Computational simulation and protein–ligand coupling approaches accelerate the discovery of new antidiabetic agents [33]. Moreover, this tool is also widely used to assist in processes, such as repositioning drugs and predicting adverse effects [34]. An exemplary application of this tool occurred during the SARS-CoV-2 pandemic, involving the utilization of molecular docking to evaluate compounds with potential antiviral properties. KM, for instance, exhibited substantial hydrogen binding to specific protein pockets in SARS-CoV-2. Molecular docking studies were used to simulate the interaction of KM with the main protease (MPro) enzymes and the angiotensin-2 converting enzyme (ACE-2). The antiviral properties of KM were confirmed through its action in these regions, highlighting its potential antiviral mechanisms [35]. Similarly, in the context of T2DM, the docking technique was instrumental in identifying the insulin receptor (IR) as a relevant target to identify natural or synthetic compounds important for the management of this disease [36].

There is limited literature examining KM’s interaction with the IR, while molecular docking studies evaluating KP’s interaction with the region of the ATP site in the IR are absent. Singh et al., 2019, found that KM exhibited increased affinity for AR receptors, but not for IR, based on docking analyses [37]. Another study explored the interaction of KM and other flavonoids with protein kinase A (PKA), revealing higher docking scores and interactions with PKA for eriodictyol, KM, and naringenin, indicating potential positive effects on PKA-dependent insulinotropic effects in vitro [38]. Yin et al. (2020) investigated KM effects from traditional Chinese medicine decoction, suggesting that KM can modulate key cellular mediators, such as AKT1, IL-6, and FOS, involved in the regulation of different biological processes, such as cell survival, the inflammatory response, among others [39]. There is currently no crystallographic model of the IR in its complete form, with the alpha and beta subunits represented together. Therefore, the subunit beta, with the most available structural chemical information, was chosen for the study’s development, which does not imply that these compounds could not interact with the alpha subunit. Our results indicate that KM has better anchorage at the ATP binding site when compared to KP, highlighting KM’s strong inhibitory potential in the insulin signaling pathway. However, assessing these compounds’ interaction with the IR α subunit was unfeasible, due to the lack of X-ray crystallography data for the α subunit, a prerequisite for molecular docking studies. Nevertheless, our work contributes specific insights into KM and KP’s anchoring in the *β* subunit of the IR.

According to van Meerloo et al., (2011), the main objective of the MTT assay is to determine viable cells, bypassing the need for extensive cell counting methods. Therefore, this technique is commonly used to assess the cytotoxicity of drugs at varying concentrations. In this assay, the mitochondrial activity in the most viable cells remains consistent, establishing a linear relationship between the number of viable cells and mitochondrial activity [40]. Acting as a positive control, DMSO at 30% concentration has been described to decrease cell viability [41]. This inhibitory effect of DMSO at 30% concentration was also observed in our study, where cell viability approaches 0% due to its cytotoxicity. For KP, there are few works in the literature evaluating its effects on cell viability, particularly its impact on HepG2 cells. Govindarasu et al. (2022) observed the cytotoxicity of KP ranging from 1 to 86 μM, in HT-29 cells, particularly noticeable from 4 μM onwards [42]. Alonso-Castro et al. (2013) determined IC50 of 86 μM for KP in HepG2 cells after 48 h [43]. However, our findings did not reveal any cytotoxic effects from KP, allowing us to proceed with the study. This study holds significance, given the limited literature on this evaluation. Considering the absence of toxicity at any assessed concentrations, the intermediate concentration of 10 μM for KP was selected for experiments studying the phosphorylation of proteins involved in the insulin signaling cascade [19,20,21].

For KM, there is little literature evaluating the effects on cell viability in HepG2 cells. Wang et al., (2018), employed concentrations ranging from 10 to 100 μM, over 72 h, revealing a proportional inhibition of cell proliferation with increasing KM concentration, particularly between 30 and 100 μM [44]. Tu et al. (2020) demonstrated that KM had a cytotoxic effect between 75 and 150 μM within the same time frame [45]. Conversely, the work by Nair et al. (2020) reported cytotoxicity across all concentrations evaluated (6.25 μM to 50 μM) with IC 50 of 9.61 μM over 72 h [46]. Several studies have explored KM cytotoxic effects in tumor cell lines (HeLa, HT-29, and A375), demonstrating similar effects at varied concentrations [47,48,49]. For instance, in RAW 264.7 cells, KM exhibited cytotoxic effects between 50 and 100 μM over 24 h [50]. However, Zhao, Yang, and Ahmad (2023) reported no decrease in cell viability in HepG2 cells treated with KM concentrations from 0 to 40 μM over 24 h [51]. Wang et al. (2003) found a concentration-dependent decrease in cell viability in prostate cancer cells with KM, noting viability levels similar to untreated cells at 10 μM. Contrary to findings in the literature, our study did not detect cytotoxic effects of KM within the range of 0.1 to 100 μM and duration time (24 h and 72 h) [52]. Despite similar methodologies, the reasons for these discrepancies require further investigation. The chosen intermediate concentration of 10 μM for KM aligns with prior studies [19,20,21].

Our findings indicate that neither KM nor KP stimulates IR and ERK1/2 phosphorylation. KP also did not stimulate AKT phosphorylation. However, KM induced AKT phosphorylation, sustaining its levels for up to 10 min post-administration. Interestingly, when compounds were administered together with insulin, no IR or ERK1/2 phosphorylation was observed. Remarkably, KM demonstrated an additive effect, increasing AKT phosphorylation levels above those triggered by insulin alone. Our initial step involved determining the optimal insulin stimulation time for the experiments, using AKT phosphorylation as a parameter [53,54]. Subsequently, we selected a 5 min insulin stimulation time frame for subsequent experiments.

Next, we focused on assessing KM and KP’s impact on insulin signaling. Insulin has two main pathways, the PI3K/PDK1/AKT pathway, which is more focused on the metabolic effects of this hormone, and the Grb-2/SOS/Ras/MAPK pathway, which is more geared toward growth effects [55]. Prior research has highlighted KM’s hypoglycemic effect in STZ-induced diabetic rats, its insulin secretion induction, antioxidant properties, and the potential to prevent atherosclerotic vascular disease [56,57]. Additionally, KM may enhance peripheral insulin sensitivity by reducing hepatic inflammation in STZ diabetes animals [5]. Conversely, limited studies have investigated KP’s effects on the insulin signaling pathway. The hypoglycemic properties of KP have been previously described [7], along with its positive impact on glucose uptake in muscle, with the possible participation of IR in this process [8]. Recently, our group demonstrated that diabetic animals treated with a *Bauhinia forficata* leaf decoction, potentially abundant in flavonoids, including KM and KP, exhibited reduced insulin resistance. This effect was markedly associated with an increase in AKT phosphorylation in skeletal muscle [58]. Our results also showed that while KM and KP did not stimulate IR phosphorylation, KM increased AKT phosphorylation for up to 10 min post-administration, independent of IR phosphorylation. Further investigations are warranted to elucidate this mechanism, especially the involvement of different AKT isoforms (AKT1, AKT2, or AKT3), given AKT2’s potential association with glucose metabolism [54]. KP, on the other hand, did not affect AKT phosphorylation.

The ERK1/2 MAPK pathway, classically not linked to insulin metabolic effects, showed no change in phosphorylation upon acute KM and KP stimulation. Even though studies by Ozaki et al. (2016) suggest MAPK’s role in energy metabolism regulation, our findings indicate no acute effects of these compounds on HepG2 cell metabolism [59]. Assessing the combined effects of KP and KM with insulin revealed no change in ERK1/2 phosphorylation levels.

Moreover, molecular docking results indicated the potential of KP and especially KM to bind the ATP binding site, potentially leading to inhibition. We are currently conducting additional experiments to evaluate the combined effect of KM and KP on IR phosphorylation. However, our current data suggest that, despite KM and KP’s inhibitory potential on the IR *β* subunit, they do not modulate IR phosphorylation. Apparently, ATP would interact more with its binding site in this subunit concerning KM and KP. Further investigation is warranted to delve into this observation.

## 5. Conclusions

In summary, our study highlights the potent insulin-mimetic impact of kaempferol in HepG2 cells, notably amplifying AKT phosphorylation, a pivotal factor in the insulin signaling cascade. This compound emerges as a promising candidate for addressing type 2 diabetes mellitus (T2DM) and exhibits interactions with cell membrane models that modify their physicochemical properties. Given the limited available data regarding KM and KP’s effects on glucose metabolism, as well as their mechanisms of action and pharmacokinetics, our work presents a compelling contribution to advancing this field.

## Figures and Tables

**Table 1:** Surface rheological parameters for monolayers submitted to 10 cycles of compression–expansion at 30 mN/m (area variation of 1% and 0.02 mHz).

	DPPC	DPPC + KP	DPPC + KM
G* (mN/m)	190.14	183.62	151.74
G′ (mN/m)	186.33	174.24	146.79
G″ (mN/m)	37.87	57.93	38.42
Θ (rad)	0.20	0.32	0.26

## Data Availability

Data are available from the corresponding author upon specific request.
